# Audiologists’ phenomenographic experiences of professional development during community service in KwaZulu-Natal, South Africa

**DOI:** 10.4102/sajcd.v69i1.844

**Published:** 2022-01-21

**Authors:** Sphilile Mbhele, Musa Makhoba

**Affiliations:** 1 Department of Audiology, Faculty of Health Sciences, University of KwaZulu-Natal, Durban, South Africa

**Keywords:** professional development, community service, work-based learning, phenomenography, graduate to professional transition

## Abstract

**Background:**

The compulsory community service programme (CSP) was implemented to improve access to healthcare and arguably facilitate the transition of graduates into independent professionals. However, its role and outcomes as a developmental platform for audiology graduates remains unclear and scant in literature.

**Objective:**

To explore the qualitative differences in the experiences of professional development among new Audiology graduates during their community service year at their fixed-placements in KwaZulu-Natal, South Africa.

**Methods:**

Within a phenomenographic design and framework, semi-structured interviews were conducted with 12 purposively sampled Community Service Officers (CSOs) of the year 2017, after obtaining ethical clearance, gatekeeper permission and participant’s consent.

**Results:**

The findings were interpreted according to the tenets of phenomenography. An outcome space based on the participants’ reported experiences, revealed three categories of description: transitioning from graduate to professional, learning in the workplace during community service and professional development. The findings reflected that the work environment, supervision, resource allocation, socialisation and infrastructure contributed to qualitatively different developmental experiences of the CSP.

**Conclusion:**

The current model of fixed-placement of the CSOs exposes them to qualitatively different developmental experiences, resulting in them attaining different developmental outcomes despite being in the same programme at the same time. Hence, we argue for an urgent CSP review, with the aim of standardising and redefining its intended outcomes and pertinent criteria for the attainment of the independent practitioner status.

## Introduction

Mandatory community service programmes (CSPs) are used globally by over 70 countries as an effective mode of providing healthcare services to underprivileged communities (Dlamini, Sekoli, & Bresser, 2019; Frehywot, Mullan, & Payne, 2010). In Africa, countries such as Mozambique and Zambia are cases in point. In Southern African countries such as Lesotho and South Africa, a 12-month period of community service is mandatory before graduates can be recognised as independent practitioners (Frehywot et al., 2010; Health Professions Council of South Africa [HPCSA], 2021). The increased burden of disease in low- and middle-income countries, shortage of qualified healthcare professionals and the uneven distribution of resources was the impetus for the CSP (Mulwafu, Ensink, Kuper, & Fagan, 2017; Olusanya, Neumann, & Saunders, 2014).

In South Africa, the CSP was introduced by the Department of Health (DoH) in 1998 for medical professions such as dentistry, medicine and pharmacy, followed by the allied health professions in 2003 including Audiology and Speech Therapy; Physiotherapy, and Occupational Therapy (Khan, Knight, & Esterhuizen, 2009; Reid, Peacocke, Kornik, & Wolvaardt, 2018). This was shortly followed by Nursing in 2008 (Govender, Brysiewicz, & Bhengu, 2015). This programme aimed to broaden access to healthcare services whilst facilitating professional development for new graduates (Reid et al., 2018). However, the latter has not received much attention in research and the field of practice; thus, the noticeable knowledge gap in this area. This study addresses this void by providing a base on which further research can be developed regarding the CSP as a developmental platform for new graduates. Through this article, we respond to this question: What are audiologists’ phenomenographic experiences of professional development during community service at their fixed-placement in KwaZulu-Natal (KZN), South Africa?

In this article, we focus on the CSP in South Africa, yet we acknowledge that issues discussed may relate to CSPs in other countries. Thus, this article contributes to what should be an international and global discussion regarding the values of the CSPs in the refinement of fit-for-purpose professionals.

The year-long CSP serves as a foundation for healthcare professionals to practice and refine their professional skills and knowledge (Beyers, 2013; Frehywot et al., 2010; Reid et al., 2018). On the contrary, Reid (2018) reported a lack of standardised guidelines for the implementation of the CSP from the National Department of Health within the South African context. The infrastructural inadequacies including poor supervision and large caseloads in public hospitals contributed to a growing dissatisfaction with the structure of the CSP (Ross, Naidoo, & Dlamini, 2018; Van Niekerk, 2012). The available literature on the CSP in South Africa (Khan et al., 2009; Reid, 2018; Ross et al., 2018; Van Niekerk, 2012) does not seem to discuss the significance of, and the need to standardise the minimum competencies for graduates to be certified as independent practitioners at the end of the CSP, particularly in audiology. The key soft and hard skills, attitudes and knowledge required for audiologists to be considered fit for independent practice seems unclear and predominantly overlooked (Reid, 2018). Thus, the supervisors of Community Service Officers (CSO’s) are likely to be left to make discretionary judgements on the readiness of the CSOs for independent practice owing to the vague community service guidelines as observed by Frehywot et al., (2010). Despite these notable shortfalls, each CSO is awarded the same certificate of independence.

The CSP ideally, should play a pivotal role in graduate development. However, the concerns regarding its unclear minimum target outcomes and indistinct guidelines are justifiable. This was the inspiration behind this study, as the authors ought to contribute an audiology perspective of such concerns. We thus, intend to facilitate a better understanding of the audiologists’ experiences of professional development during the CSP, which has been a largely neglected area of audiology. Whilst literature reports on professional development, as seen in a 15-year review study by Reid et al. (2018) on CPS for medical doctors in South Africa, it is limited in its exploration of the qualitative differences in professional development and their respective impact on readiness for independence practice.

The fixed-placement model of CSOs in South Africa does not allow CSOs to rotate across different sites, which according to Ross et al. (2018) brings about risks of inconsistency of experiences, skills and knowledge gained by the medical interns. The absence of a similar study in the field of audiology, arguably, suggested that the impact of the fixed-placement model of audiology CSOs on their developmental experiences compared with that of rotating across different CSP sites remained a mystery. This was our primary concern. In Medicine, the HPCSA extended the internship period and stipulated that rotations between clinical sites were mandatory to ensure adequate preparedness and competency in graduates (Nkabinde, Ross, Reid, & Nkwanyana, 2013). Therefore, we argued that the consequences of the fixed-model of placement in audiology needed to be well researched and critically reviewed in order to devise appropriate, field-specific interventions in response to the aforementioned challenges.

This article is the first of two based on a masters research project (Mbhele, 2019). Through this article, we report on CSOs’ experiences of community service in KZN within the year 2017. We also discuss the differences and the factors that contributed to those differences in their experiences. We thus, present an audiology perspective to the challenges of the CSP fixed-placement model, to supplement those observed by the aforementioned disciplines. The subsequent article builds on this study’s findings as a foundation to propose possible changes to the current model of CSO placement, to address the reported challenges.

## Methodology

### Design and framework

Phenomenography was adopted as the study framework and design. It guided the conceptual understanding of CSOs’ experiences and the adopted study methods. Phenomenography is a study of qualitatively different ways in which people can experience and understand the same phenomena (Marton, 1981). It is located within an interpretive paradigm as it is underpinned by the assumption that the world exists as perceived by those who live in it, and two or more individuals exposed to the same phenomenon, will have qualitatively different experiences of it (Han & Ellis, 2019; Ireland, Neofa, & Harding, 2009; Marton, 1981). Therefore, as a study frame, phenomenography provided a lens through which the audiology CSOs’ developmental experiences during their community services (CS) were explored.

Phenomenographic experiences as they exist in the consciousness of those who lived them are either referential or structural (Marton & Pong, 2005). The former refers to the meaning attributed to the experiences, whilst the later refers to the aspects of the experience (Han & Ellis, 2019; Marton & Pong, 2005). Applied to this study, the referential aspects pertained to what the experiences of community service meant to the CSOs. The structural aspect pertained to the elements of the CSP that impacted the different experiences. The structural aspect comprised an internal horizon, referring to the relationship between the phenomenon and its various parts and an external horizon, which refers to the foundation of the experience (Yates, Partridge, & Bruce, 2012). In this study, the undergraduate audiology programme at the respective universities was the foundation of the experiences and was largely supported by work-integrated learning methods. As CSOs’ interacted with the work context, which is inclusive of hospital staff, patients, clinical procedures and the administrative processes, their experiences of the programme were shaped. The methods of data generation and processing were developed within the aforementioned frame.

### Methods

The study was conducted in KZN, South Africa because of convenience and feasibility of the data generation process that did not compromise the quality of data generation. Gatekeeper permission to conduct the study in KZN was sought via email from the Directorate of Health Research and Knowledge Management, the Assistant Director of Disability and Rehabilitation online application for the gatekeeper permission. The study commenced following the attainment of full ethical clearance from the university the researcher was registered with. All 28 audiology graduates, placed in KZN across the different municipalities in 2017 (Table 1) were invited telephonically to participate in the study. They were contacted through their institution contact numbers, which were publicly available. The list of all CSOs was publicly accessible from the Department of Health Website. A total of 12 CSOs agreed to participate; a participant number that was sufficient and consistent with the phenomenolgraphy design (Tight, 2016). Each participant had qualified with a bachelor’s degree in audiology in the year 2016. Two of the 12 participants participated in the pilot study, their responses were included in the main study as minimal amendments were made to the pilot study, rendering their response relevant in the main study. The participants’ biographical information is presented in Table 1.

**TABLE 1:** Participants’ biographical information and placement in various districts in KwaZulu-Natal.

Biographical information	Subheading	No. of participants
Gender	Male	2
	Female	10
Geographical area(district)	Ugu	2
	Zululand	2
	Mkhanyakude	2
	Uthukela	1
	King Cetshwayo	1
	Amajuba	2
	Ilembe	1
	Umzinyathi	1
Hospital facility	District hospital	8
	Regional hospital	2
	District and regional hospital	2

The participants were predominately female, and they all completed their CS in rural communities. This was consistent with the demographics of the CSOs at the time of the study, as the profession was and is still female dominated (Du Plessis, 2018). Targeting representative participants contributed to strengthening the contextual validity of the study’s finding (Dikilitaş & Griffiths, 2017).

### Data generation and tools

An interview schedule was developed by the researcher, informed by relevant literature (Åkerlind, 2005; Rands & Gansemer-Topf, 2016; Reed, 2006; Strydom, 2013). The interview schedule comprises three sections, including the background, experiences of CS and professional development, supplemented by probing questions. All these elements allowed for the generation of phenomenographic data that focused on the holistic and qualitatively different experiences (Han & Ellis, 2019; Marton & Pong, 2005). Key information about the participants’ role and rights were discussed, then shared through the information document before each participant signed the informed consent form. All interviews were conducted in English and each interview ranged from 25 min to 60 min, which all participants were comfortable with.

### Data analysis

Each audio-recorded interview was transcribed verbatim (Stuckey, 2014) and uploaded into NVivo (version 11) for analysis. Through content analysis, data were manually organised into key units of meaning in the form of words or phrases related to the phenomenon and the tenants of the adopted conceptual frame (Erlingsson & Brysiewicz, 2017). The codes were collated into themes, reflecting the different ways in which CSOs experienced development during their CS year (Reed, 2006; Ireland et al., 2009). The participants’ conceptions were arranged according to their similarities and differences and then placed into categories according to their core meaning (categories of description) (Han & Ellis, 2019). An outcome space was then constructed, where the conceptions were categorised and the relationships between the categories were defined (Barnard, McCosker, & Gerber, 1999; Yates et al., 2012). Therefore, in this article, the outcome space represents the qualitatively different ways professional development was experienced by CSOs in 2017.

### Quality of the data

The aforementioned ethics committees’ approvals of the study ensured that it would be ethically and scientifically sound. Trustworthiness of the research findings was maintained through ensuring credibility, dependability and transparency (Anney, 2014). Selection of representative sample ensured that the data were contextually valid (Dikilitaş & Griffiths, 2017). The excerpts from the interviews were shared to ensure transparency (Anney, 2014). The pilot study allowed for the necessary refinements in the data generation processes that could compromise the quality of the study (Malmqvist, Hellberg, Möllås, Rose, & Shevlin, 2019). All possible challenges were resolved, eliminating ambiguities between researchers and participants. Methodological rigour allowed for an in-depth exploration of the qualitative differences in CS enhanced transparency, ensuring that the findings are dependable (Anney, 2014).

### Ethical considerations

The ethical approval and gatekeeper permission were sought and granted by the University of KwaZulu-Natal’s Human and Social Sciences Ethics Committee (HSS/1040/108M), and the Department of Health (KZ-201809-023), respectively, before the study commenced. Participants signed informed consent forms before participating. The researcher abided by the World Medical Association’s Helsinki Declaration of research ethical principles for medical research involving human subjects (World Medical Association, 2013).

## Results

**FIGURE 1:**
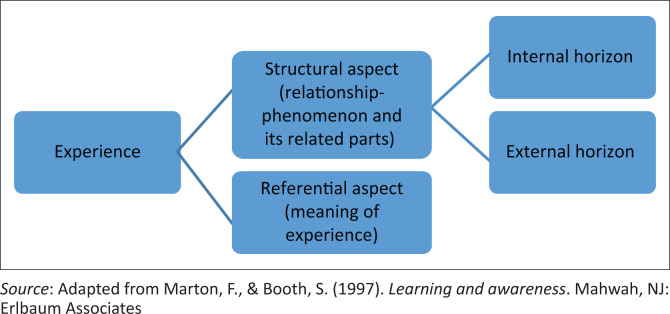
Illustration of the characteristics of an experience.

The findings are organised according to the conceptual framework (Figure 1) and presented in the outcome space (Table 2), which illustrates the categories of descriptions, conceptions and key differences of how CSOs conceptualised their lived experiences of CS.

**TABLE 2:** Outcome space of the participants’ experiences.

Aspect		Category	Conceptions	Key differences
Referential aspects		Transitioning from graduate to professional is challenging.	Conceptions of transition and adaptation to the workplace	Better learning opportunities during CS versus better learning opportunities in undergraduate training
Structural aspects	External horizon	Learning in the workplace during CS	Conceptions of CS as a work context	Work environment The impact of resources in the practice of audiology Supervision and support
	Internal horizon	Professional development	Conceptions of knowledge development	Experiences of practical knowledge development Experiences of experiential knowledge development Experiences of theoretical knowledge development

CS, community service.

The findings indicate that there are differences in the CS experiences and the conceptions of CSP amongst participants. Participants expressed differences in the work context, supervision support and space of professional development. These results have been reported on a Masters research project conducted by Mbhele (2019), therefore, a brief overview will be presented in this section to illustrate the variations in the developmental outcomes stemming from one programme.

### Transitioning from graduate to professional (the referential aspect)

There were no noticeable qualitative differences in the experiences of transitioning from graduate to professional during the CS year. However, the participants’ experiences were significant in understanding the developmental role of CS, thus it is worth reporting. Participants expressed that transitioning into the CSP was initially difficult, as they had to deal with a surge of emotions, constructing a professional identity and the pressures of the work environment. Specifically, the c*onceptions of transition and adaptation to the workplace* were identified for this category, as discussed in the next subsection.

#### Conceptions of transition and adaptation to the workplace

Participants reported experiencing emotions such as anxiety, stress, frustration and exhaustion related to transitioning to a working place. Some of these feelings were further exacerbated by factors such as large patient caseloads, relocation, work conflict and not having supervisors. These are amongst the external factors that contributed to the qualitative differences in experiences. From the responses, it was evident that the participants were able to develop emotional maturity and coping mechanisms during their CS in order to effectively adjust to their work environment. They reported feeling more resilient and more self-motivated as the year progressed. This is seen in the following excerpts:

‘So, I think, as I said I was very nervous at first and then you got to a point where you were bit more confident, a bit more relaxed.’ (Participant 6, 25 years old, Female)

The participants also reported that their transitional experiences were also influenced by the noticeable differences between their previous learning environment and the workplace. They described their undergraduate training as a support-rich environment that provides ample resources for learning. Conversely, other participants felt that CS gave more opportunity for learning and development than their previous tertiary institution as detailed in the following excerpts:

‘I think in terms of knowledge of different diagnosis, different pathologies that’s much better. Also, just skills like I picked up much better. Like in campus I had hardly done any ear moulds. I did like 2 the whole year, I only did like 3 for my whole 4 years and now I did like 20 or 30, I don’t know how many. So, things like that […].’ (Participant 9, 24 years old, Female)

‘Yeah, because in varsity it was more following the rules whereas in comm-serve I got to do my own thing and I got to understand how I work and the people that I saw who developed my own style of doing things.’ (Participant 2, 24 years old, Female)

The given excerpts are some of many examples indicating the effectiveness of the CSP as a developmental platform for new audiology graduates. Thus, participants learnt a lot about different aspects of working as a professional, as indicated in the following section, which added to their professional development as an outcome of being through the CSP.

### Learning in the workplace during CS (structural aspect-external horizon)

According to Strode (2010) the work context has influence over professional learning and development; be it negative or positive. Thus, the explored *Conception of CS as a work context* provides a deeper understanding into factors that affect professional development of COSs. Key differences in the work environments, supervision and the availability of resources amongst CSOs were identified and are further discussed in relation to the participants’ conceptions.

#### Work environment

There were variations in the participants’ conceptions of the impact of their work environment in their development. Half of the participants indicated their work environment had a positive impact on their learning and this was facilitated by availability of supervision, feeling welcomed and socialisation as seen in this excerpt:

‘My supervisor was like probably, one of the most amazing, most amazing woman I ever met and her co-worker um also she Xhosa, also incredible people. […]. You know that type of … So, she also kind of made it relaxed and you know, I remember I used to offer to clean all the probes. You know like I’ll clean all the probes for you…. She was like “Sit down, we’re all in this together. We are a team. Like, I can learn from you as well”.’ (Participant 10, 26 years old, Female)

Others reported starting up an audiology department without supervision and work conflict to have negatively impacted their development in the workplace. Furthermore, they indicated that challenges with hospital management and negative attitudes towards audiology services posed as limitations to their practice of Audiology as seen in these excerpts:

‘[*Y*]eah as much and it might have to do with limited understanding and yeah that teamwork within the TB clinic wasn’t very good. But other than that (site where participant worked) did actually have quite good resources in terms of audiology, we had a big budget for hearing aids and yeah, it was a good experience despite, yeah. Management within the hospital didn’t always make it easy, that and how the whole hospital is managed also the things like ordering equipment or ordering stock or staff like that.’ (Participant 7, 25 years old, Female)

Equally, some participants observed that it was such challenges that facilitated the development of leadership skills, self-assurance and independence, as explained by the following excerpt:

‘[…] Sometimes it is something that you have to, I’m trying to find right words to explain this, like you get patients that you see that okay, if I’m going to do this, this and this according to what I was taught in varsity, it won’t work. So, you get to find your own way of doing things but which doesn’t violate the rules but you get to find your own way of dealing with situations.’ (Participant 4, 25 years old, Male)

These findings highlight the differences in the experiences of the CSOs, which supports the key point of concern and further necessitates the argument for change as expressed in the discussion section.

#### The impact of resources in the practice of audiology

Participants were exposed to a new clinical environment that is notably different from their tertiary institutions. They reported the importance of resource availability in their experiences of professional development. They reported experiences that indicated variations in what CSOs considered as limited resources. Factors such as staffing, equipment, management and finances were observed as resources, which were viewed as unevenly distributed within the healthcare context.

Some participants reported a lack of audiological equipment in the department whilst some reported limited budget allocation for equipment acquisitions. For others, the available equipment was uncalibrated, thus limiting its use. Some participants only had access to basic audiometric testing equipment whilst others only lacked the advanced (electrophysiological) equipment. This and other challenges such as restricted work space impacted negatively their development, limiting their learning and clinical practice as seen in the following excerpts:

Participant 6: ‘And I think better in the sense that also equipment related because our ABR (Auditory brainstem responses, objective test of auditory function, Katz et al., 2015) was broken in the first three quarters of the year. So, if that (the ABR equipment) was working…, well I don’t do ABR now so that would have been an amazing experience.’ (Participant 6, 25 years old, Female)

‘Audiometry I could have developed but I couldn’t because of equipment, resources shortage and as much as we were kind of exposed to screening in a noisy environment, how to reduce the noise to signal ratios and all that what you learn in varsity. I could have as well there because we had clinics which were very busy and had limited space.’ (Participant 3, 24 years old, Female)

Conversely, some participants expressed that they had sufficient resources to foster their learning as seen in these excerpts:

‘Its just me. Ok…like I said, it (site where the participant worked) was well resourced. I got the opportunity to use most of the theory that I learnt and used it theoretically, practically….’ (Participant 12, 24 years old, Female)

These findings indicate how the differences in the availability of resources contributed in shaping the level of exposure to different audiological practices. This brings to question the extent of variation in the CSP contribution towards developing and refining the clinical and professional skills of the new graduates, particularly across different placement sites.

#### Support and supervision

This aspect of the finding highlights the discrepancy in support and supervision received by CSOs in the various healthcare institutions. Some participants indicated receiving adequate supervision, which facilitated independence, confidence and clinical skills as seen in this excerpt:

‘[*Y*]eah so my supervisors were always very helpful and very nice to me and understanding so that was, that was nice and I think that made the difference, that if you have nice supervisors the experience there is much different. So if they sort of… They…, You know that’s your supervisor but they kind of treat you like equals. That’s what I’m saying. They respect you and you respect them. So that’s what I like […].’ (Participant 9, 24 years old, Female)

Other participants expressed limited or no supervision, which facilitated the need to learn discipline, self-reliance and operational and patient management skills. This affected their development throughout the year as seen in this excerpt:

‘It was a good and bad experience in that I learnt to be more independent and I learnt to be more motivated because I didn’t have anybody to rely on so I was always trying to push myself to do better and it was negative because I didn’t have anybody I could rely on so and whenever I wanted to do something different I didn’t have anybody backing me up so I was always just alone.’ (Participant 2, 24 years old, Female)

A few of the participants reported receiving intermittent supervision, which alleviated feelings of stress during CS. This was observed as a significant hindrance to their development.

‘[*U*]hm, more likely of mixed emotions because earlier I had a supervisor and you know in areas where I wasn’t sure, I would run to my supervisor all the time. And then as time went on and then I didn’t have a supervisor because she was sick and then I got this one incident I got a very stressful patient and then I was very emotional and I couldn’t handle it […].’ (Participant 11, 25 years old, Female)

Again, this brings to question the extent to which availability of supervision is regulated in order to have it relatively standardised across the different CS placement sites. However, the findings have indicated room for improvement in this regard.

### Professional development (structural aspect-internal horizon)

The work environment afforded by the CSP was developmental to CSOs, as previously observed by Wranz (2011) and Beyers (2013). The participants in this study reported having experienced practical and experiential knowledge in various areas of clinical practice and professional conduct during their CS year, including audiological skills such as cerumen management, basic audiometry, ototoxicity monitoring and patient-management. They indicated that daily repetition of procedures, high caseload and variety of cases improved their clinical judgement, confidence and decision-making skills, as seen in the next excerpt:

‘I think it increases your confidence quite a bit and I was quite fortunate that last year to have seen quite a large patient load and it does certainly give you confidence in your testing procedures, more confidence in your ability to diagnose hearing and related disorders […].’ (Participant 1, 30 years old, Male)

However, other participants felt there was not much improvement in their theoretical knowledge and CS served to cement theory obtained during their undergraduate training. Theoretical knowledge was reported to have been an overlooked part of CS. However, activities such as presentations, journal clubs and workshops were used by CSOs to develop their theoretical knowledge as expressed in the following excerpt:

‘I won’t lie. I think because there was no supervisor involved from my side, there was no learning or reading up on anything new in terms of theory so I can’t…anything stands out that I can say I learnt. […] in terms of workshops and CPD courses, it was a very neglected area.’ (Participant 12, 24 years old, Female)

Granted, all participants experienced professional development, as previously indicated, differences were observed in the type of knowledge developed and the extent of the professional development. The different contextual factors identified in the participants’ conceptions were noticed to have contributed to the qualitative differences in the CSOs’ experiences of audiology in 2017.

## Discussion

Guided by the phenomenography as a framework, the primary focus of this study was to highlight the key variations in audiology CSOs’ developmental experiences. The identified referential aspect of the phenomenographic experiences indicated transitional challenges experienced by all participants, which was predominantly emotionally challenging, as the findings indicated. These findings were consistent with previous research (De Villiers, Van Heerden, & Van Schalkwyk, 2018). De Villiers et al. (2018) qualitative, longitudinal study explored medical intern supervisors’ views on internship training in South Africa. The study found that transitional challenges were common amongst medical interns, including difficulties with applying theory to practice and heeding the work environment demands such as long hour shifts, which are emotionally stressful.

This study’s findings suggest that there are three qualitatively different ways in which the professional development can be conceived and that the outcomes of CSP are largely affected by factors such as CSO site placement and work environment, the transition into the workplace and availability of both human and clinical resources. Currently, it appears that the main focus of CSP is for service delivery, where professional development has not been largely prioritised. As a compulsory developmental platform prior to certification for independent practice, it is, arguably, fair to expect the CSP to provide a conducive environment for CSOs to develop in all areas related to their scope of practice. However, the findings indicate that this was not the case. The fixed-placement of CSOs at the DoH sites facilitated the heterogeneity of experiences as there were CSOs who received minimum exposure and those who had exposure to a wide spectrum of audiological practices. Furthermore, supervision was another key factor where some participants had onsite audiology supervisors whilst others did not. Support and resource availability have been reported as paramount contributing factors in service provision and the development of CSO in dentistry (Machete, Makwakwa, Moipolai, & Motloba, 2021). These were reported by these authors in a correctional study conducted with Dentists who had completed their community service in South Africa. In audiology, similar challenges were reported over a decade ago by Khan et al. (2009), and yet, for some participants, this was still a challenge in the year 2017.

The community service supervisors have a significant influence in the kind of experiences each CSO is exposed to Shezi (2014). At least this was the case in nursing, according to Shezi (2014). Ross et al. (2018) evaluating the medical internship programme at a public hospital in South Africa in 2016, revealed that the level of supervision, consistency of mentorship and training received by interns at public institutions were a cause for concern. This was further confirmed by the HPCSA internship subcommittee that identified gaps in supervision and training of graduates (Ross et al., 2018). The availability of an onsite CS supervisor has not been a full requirement of the CS placement criteria (Reid, 2001), even though it appears to be a strong consideration since 2018 according to the Internship and Community Service Placement guidelines for 2017–2018 (South African National Department of Health, 2018).

Availability of resources is another crucial factor that plays a role in developing competencies for audiological practice. Some participants reported lack of basic audiological equipment, availability of broken and uncalibrated equipment and ultimately, inadequate infrastructure for clinical practice, which poses limitations on the clinical skills developed during the CSP. Similar concerns were raised by Matlhaba, Pienaar and Sehularo (2019) studying the nurse’s clinical competence in some hospitals in the North West Province, South Africa. Regardless of these factors, the assumption is that the certification of independent practice at the end of CS should signify competency in all or most of audiology areas of practice. Thus, we remain concerned with the variations in the CS experiences and possible implications it could have on the effectiveness of the same CSP in preparing different CSOs for independent practice. This concern is worsened by the absence of any board exams or competency assessment measures that would at least document the level of or preparedness for independent practice or lack thereof at the end of the CSP for each CSO.

Whilst the Audiology undergraduate programme offers intensive clinical exposure, one could argue that it does not negate the need for further professional development that takes place during an in-service or CSP. The findings of this study indicated that CSP does facilitates professional development post-graduation as was indicated by Govender et al. (2015), and Van Stormbroek and Buchanan (2016). However, the development of CSOs seems to occur haphazardly with little support and structure in practice to ensure that standardised developmental outcomes are met across the programme. Similarly, we recognise the developmental value of infrastructural constraints within the context of CSP. Reid et al. (2018) stated that the human, financial, equipment and other resource constraints contribute to the development of resilient and adaptable professionals. Our key concern is the degree of variation in developmental experiences in the same group of CSOs resulting from these constraints. This concern is not new in audiology as Khan and colleagues reported similar concern with resource constraints in health sciences rehabilitation fields, including audiology (Khan et al., 2009).

Perhaps to remediate the present challenges in the programme, strong considerations for the introduction of an in-service training and internships programme in audiology followed by a formalised standardised assessment of competency, need to be made. Similar strategies have been utilised in undergraduate healthcare programmes such as pharmacy and medicine, whilst others don’t (Opoku, Van Niekerk, & Jacobs-Nzuzi Khuabi, 2020). Audiology, occupational therapy, physiotherapy and optometry are a case in point. It is not clear if the CSP negates the need for in-service training in these fields. In pharmacy, graduates are required to complete 1 year of internship, submit continuing professional development (CPD) portfolio, their supervisor submit a progress report with areas of competence and they are then required to successfully complete a pre-registration examination prior to being registered as a Pharmacist (The South African Pharmacy Council, 2020). Furthermore, they are required to involve themselves in community work and community outreach to gain experience and interpersonal skills (Farzadeh, 2019). Similarly, the medicine profession has a 2-year internship programme, to allow for graduates to acquire all the necessary skills prior to starting with the CSP (Van Niekerk, 2012). This incorporates clinical rotations, which optimised each intern’s exposure across different areas of practice, enhancing their knowledge and skills (Nkabinde et al., 2013; Reid et al., 2018). The CS programme was viewed by the majority of the medical CSOs as an effective developmental platform in the study conducted in South Africa.

In practice the guidelines, including the outcome measures of the CSP, as a developmental platform remain unclear (Reid, 2018). We, therefore, argue that seemingly subjective, discretionary judgement of the CSOs readiness to practice independently requires a review. The differences in CSOs experiences suggests that if the benchmark for minimum criteria of independent practice skills outcome exists in audiology, its use differs across the CS sites, and that there is likely too much reliance, in practice, on the subjectivity of the CS supervisors. We thus, argue that the lack of, or limited (practical application of the) *standardisation* and *benchmarking* of the CSP, should be amongst the highly prioritised concerns and reasons for the review of the current CSP. We elaborate on this argument in the subsequent article.

### Limitations

Methodologically, the participants’ reflection has to be taken as true, without the necessity to control for untruthful reflections. It is possible that the participants’ reflections could have left out some important aspects of their experiences, despite extensive probing. It would have been of benefit to conduct member checking and reflections to verify the accuracy of the interpretation of the participants’ responses. However, time and other challenges did not allow for this aspect to be realised. Lastly whilst the trustworthiness of the findings remains strong, in our opinion, it would have been even stronger had the findings been analysed by two or more researchers initially. But the study being at a master’s level limited us from doing so.

## Conclusion and recommendations

The CSOs experiences of professional development demonstrate the various ways in which they interacted with work environment, staff and the community as a whole through the CSP. The conceptions discovered in this study reveal the close relationship between the nature of CSP context and the developmental outcomes it intends on producing as it influences the development of skills, knowledge and professional autonomy.

This research study further highlights the limitations of using the fixed-placement model within the CSP as optimises variations in developmental outcomes and demonstrates a lack of standardisation in the evaluation criteria that declares the readiness for independent practice. The study argued for an urgent review of this model and majority of the challenges within the programme warrant an urgent response. Thus, we provide suggestions for improvements in the subsequent article. In addition, the variations of experiences reflected in this study should serve as a foundation for further research and a broader discussion for the improvement of the programme and its intended outcomes.

## References

[CIT0001] Åkerlind, G.S. (2005). Variation and commonality in phenomenographic research methods. *Higher Education Research & Development*, 24(4), 321–334. 10.1080/07294360500284672

[CIT0002] Anney, V.N. (2014). Ensuring the quality of the findings of qualitative research: Looking at trustworthiness criteria. *Journal of Emerging Trends in Educational Research and Policy Studies*, 5(2), 272–281.

[CIT0003] Barnard, A., McCosker, H., & Gerber, R. (1999). Phenomenography: A qualitative research approach for exploring understanding in health care. *Quality Health Research*, 9(2), 212–226. 10.1177/104973299129121794 10558364

[CIT0004] Beyers, B. (2013). *Experiences of community service practitioners who are deployed at a rural health facility in the Western Cape.* Western Cape: University of the Western Cape.

[CIT0005] De Villiers, M., Van Heerden, B., & Van Schalkwyk, S. (2018). ‘Going the extra mile’: Supervisors’ perspectives on what makes a ‘good’ intern. *South African Medical Journal*, 108(10), 852–857. 10.7196/SAMJ.2018.v108i10.13052 30421714

[CIT0006] Dikilitaş, K., & Griffiths, C. (2017). Thinking about the context: Setting (where?) and participants (who?). In *Developing language teacher autonomy through action research* (pp. 89–105). Cham: Palgrave Macmillan.

[CIT0007] Dlamini, L., Sekoli, L., & Bresseret, P. (2019). Perceptions and short-term experiences of newly qualified radiographers performing compulsory community service. *Radiography*, 25(2), 108–113. 30955682 10.1016/j.radi.2018.11.003

[CIT0008] Du Plessis, S. (2018). Male students’ perceptions about gender imbalances in a speech-language pathology and audiology training programme of a South African institution of higher education. *The South African Journal of Communication Disorders*, 65(1), e1–e9. 10.4102/sajcd.v65i1.570 PMC5968872 29781703

[CIT0009] Erlingsson, C., & Brysiewicz, P. (2017). A hands-on guide to doing content analysis. *African Journal of Emergency Medicine*, 7(3), 93–99. 10.1016/j.afjem.2017.08.001 30456117 PMC6234169

[CIT0010] Farzadeh, S. (2019). The role of community service in building better pharmacists. *American Journal of Health-System Pharmacy*, 76(10), 644–645. 10.1093/ajhp/zxz037 30934059

[CIT0011] Frehywot, S., Mullan, F., & Payne, P.W. (2010). Compulsory service programmes for recruiting health workers in remote and rural areas: Do they work? *World Health Organization*, 88, 364–370. 10.2471/BLT.09.071605 PMC2865657 20461136

[CIT0012] Govender, S., Brysiewicz, P., & Bhengu, B. (2015). Perceptions of newly qualified nurses performing compulsory community service in KwaZulu-Natal. *Curationis*, 38(1), 1–8. 10.4102/curationis.v38i1.1474 PMC6091806 26244458

[CIT0013] Han, F., & Ellis, R. (2019). Using phenomenography to tackle key challenges in science education. *Frontiers in Psychology*, 10, 1414. 10.3389/fpsyg.2019.01414 31293478 PMC6603223

[CIT0014] Health Professions Council of South Africa (HPCSA). (2021). *Legal and regulatory affairs.* Health Professions Council of South Africa. Retrieved from https://www.hpcsa.co.za/Uploads/Legal/legislation/health_professions_ct_56_1974.pdf

[CIT0015] Ireland, J.T., Neofa, Z., & Harding, T. (2009). *The tale of four researchers: Trials and triumphs from the phenomenographic research specialization*. Brisbane: Queensland University of Technology.

[CIT0016] Katz, J., Chasin, M., English, K.M., Hood, L.J., & Tillery, K.L. (Eds.). (2015). *Handbook of clinical audiology (Seventh)*. New York: Wolters Kluwer Health.

[CIT0017] Khan, N., Knight, S., & Esterhuizen, T. (2009). Perceptions of and attitudes to the compulsory community service programme for therapists in KwaZulu-Natal. *The South African Journal of Communication Disorders*, 56(1), 17–22. 10.4102/sajcd.v56i1.189 20235490

[CIT0018] Machete, M.L., Makwakwa, L.N., Moipolai, P.D., & Motloba, D.P. (2021). Compulsory community service for dentists – Opportunity for meaningful reform. *South African Dental Journal*, 76 (4), 201–206. 10.17159/10.17159/2519-0105/2021/v76no4a4

[CIT0019] Malmqvist, J., Hellberg, K., Möllås, G., Rose, R., & Shevlin, M. (2019). Conducting the pilot study: A neglected part of the research process? Methodological findings supporting the importance of piloting in qualitative research studies. *International Journal of Qualitative Methods, 18,* 1–11. 10.1177/1609406919878341

[CIT0020] Marton, F. (1981). Phenomenography: Describing conceptions of the world around us. *Instructional Science*, 10, 177–200. 10.1007/BF00132516

[CIT0021] Marton, F., & Booth, S. (1997). *Learning and awareness*. Mahwah, NJ: Erlbaum Associates.

[CIT0022] Marton, F., & Pong, W.Y. (2005). On the unit of description in phenomenography. *Higher Education Research & Development*, 24(4), 335–348. 10.1080/07294360500284706

[CIT0023] Matlhaba, K.L., Pienaar, A.J., & Sehularo, L.A. (2019). Community service nurses’ experiences regarding their clinical competence. *Health SA Gesondheid* 24(0), a1284. 10.4102/hsag.v24i0.1284 PMC6917431 31934440

[CIT0024] Mbhele, S. (2019). *The professional knowledge development of Community Service Audiologists in KwaZulu-Natal- A phenomenographic study*, Unpublished Master’s dissertation, University of KwaZulu-Natal, Durban. 10.4102/sajcd.v69i1.844 PMC8831927 35144439

[CIT0025] Nkabinde, T.C., Ross, A., Reid, S., & Nkwanyana, N.M. (2013). Internship training adequately prepares South African medical graduates for community service – With exceptions. *South African Medical Journal*, 103(12), 930–934. 10.7196/SAMJ.6702 24300632

[CIT0026] Olusanya, B.O., Neumann, K.J., & Saunders, J.E. (2014). The global burden of disabling hearing impairment: A call to action. *Bulletin of the World Health Organization*, 92(5), 367–373. 10.2471/BLT.13.128728 24839326 PMC4007124

[CIT0027] Opoku, E.N., Van Niekerk, L., & Jacobs-Nzuzi Khuabi, L.A. (2020). Exploring the factors that affect new graduates’ transition from students to health professionals: A systematic integrative review protocol. *BMJ Open*, 10(8), e033734. 10.1136/bmjopen-2019-033734 PMC7402005 32747347

[CIT0028] Rands, M., & Gansemer-Topf, A.M. (2016). Phenomenography: A methodological approach for assessment in student affairs. *Education Publications*, 45 Retrieved from https://lib.dr.iastate.edu/edu_pubs/45

[CIT0029] Reed, B.I. (2006). Phenomenography as a way to research the understanding by students of technical concepts. *Núcleo de Pesquisa em Tecnologia da Arquitetura e Urbanismo (NUTAU)*. Sao Paulo: Technological Innovation and Sustainability.

[CIT0030] Reid, S. (2018). South African health review 2018. In L.C. Rispel & A. Padarath (Eds.), *20 years of community service in South Africa: What have we learnt?* (pp. 41–50). Durban: Health Systems Trust. Retrieved from http://www.hst.org.za/publications/Pages/SAHR2018

[CIT0031] Reid, S.J. (2001). Compulsory community service for doctors in South Africa – An evaluation of the first year. *South African Medical Journal*, 91(4), 329–336. 11402906

[CIT0032] Reid, S.J., Peacocke, J., Kornik, S., & Wolvaardt, G. (2018). Compulsory community service for doctors in South Africa: A 15-year review. *South African Medical Journal*, 108(9), 741–747. 10.7196/SAMJ.2018.v108i9.13070 30182899

[CIT0033] Ross, A., Naidoo, S.S., & Dlamini, S. (2018). An evaluation of the medical internship programme at King Edward VIII hospital, South Africa in 2016. *South African Family Practice*, 60(6), 187–191. 10.1080/20786190.2018.1504866

[CIT0034] Shezi, B. (2014). *The needs of community service nurses with regard to supervision and clinical accomponiment*. Potchefstroom: North-West University.

[CIT0035] South African Nation Department of Health. (2018). *Internship and Community Service Placement Guidelines for 2017/18*. Pretoria: NDoH.

[CIT0036] Strode, A. (2010). Students’ independent professional activity in pedagogical practice. *Journal of Teacher Education for Sustainability*, 12, 38–58. 10.2478/v10099-009-0053-y

[CIT0037] Strydom, H. (2013). An evaluation of the purposes of research in social work. *Social Work Journal*, 49(2), 149–164. 10.15270/49-2-58

[CIT0038] Stuckey, H.L. (2014). The first step in data analysis: Transcribing and managing qualitative research data. *Journal of Social Health and Diabetes*, 2(1), 6–8.

[CIT0039] The South African Pharmacy Council. (2020). *Registered persons*. South African Pharmacy Council. Retrieved from https://www.pharmcouncil.co.za/Intern_Year

[CIT0040] Tight, M. (2016). Phenomenography: The development and application of an innovative research design in higher education research. *International Journal of Social Research Methodology*, 19(3), 319–338. 10.1080/13645579.2015.1010284

[CIT0041] Van Niekerk, J.P. (2012). Internship and community service require revision. *South African Medical Journal*, 102(8), 638. 10.7196/SAMJ.6094 22831924

[CIT0042] Van Stormbroek, K., & Buchanan, H. (2016). Community service occupational therapists: Thriving or just surviving? *South African Journal of Occupational Therapy*, 46(3), 63–72. 10.17159/23103833/2016/v46n3a11

[CIT0043] World Medical Association. (2013). World Medical Association declaration of Helsinki – Ethical prinicples for medical research involving human subjects. *JAMA: Special Communication*, 310(20), 2191–2194. 10.1001/jama.2013.281053 24141714

[CIT0044] Wranz, E. (2011). *Compulsory community service for speech-language and hearing therapy professionals: Readiness, reality and readjustment*. Unpublished Master’s thesis, University of Stellenbosch, Cape Town. Retrieved from http://scholar.sun.ac.za/handle/10019.1/6599

[CIT0045] Yates, C., Partridge, H., & Bruce, C.S. (2012). Exploring information experiences through phenomenography. *Library and Information Research*, 36, 96–119.

